# California

**Published:** 1896-10

**Authors:** 


*California.* Riverside, through its health-officers, has taken up the inspection of the dairy-herds with a view of eliminating all tuberculous animals. The *News* calls upon every citizen to assist in the crusade against tuberculosis in dairy-cows, by refusing to buy milk unless the dealer can display a clean bill of health, which advice other journals in all cities might give to their readers.

One of San José's herds of cattle, which was recently a source of the existence of tuberculosis, was again tested September 8th, and of the thirty-four animals none were found diseased.

In the same county, Santa Clara, some condemned cattle were surreptitiously sold, but were subsequently recaptured and slaughtered.

Drs. Pierce and Archibald have an excellent article in the Oakland *Enquirer* on the subject of tuberculosis and its relations in regard to the milk- and meat-supply of large cities.

